# Prognostic Value of Cardiac Magnetic Resonance in Assessing Right Ventricular Strain in Cardiovascular Disease: A Systematic Review and Meta-Analysis

**DOI:** 10.31083/j.rcm2312406

**Published:** 2022-12-12

**Authors:** Kairui Bo, Zhen Zhou, Zhonghua Sun, Yifeng Gao, Hongkai Zhang, Hui Wang, Tong Liu, Lei Xu

**Affiliations:** ^1^Department of Radiology, Beijing Anzhen Hospital, Capital Medical University, 100029 Beijing, China; ^2^Discipline of Medical Radiation Science, Curtin Medical School, Curtin University, 6845 Perth, Western Australia, Australia; ^3^Department of Cardiology, Beijing Anzhen Hospital, Capital Medical University, 100029 Beijing, China

**Keywords:** meta-analysis, strain, right ventricle, cardiac magnetic resonance, prognosis

## Abstract

**Objective:**

To evaluate the prognostic value of cardiac magnetic resonance (CMR) imaging in assessing right ventricular strain via meta-analysis of current literature.

**Background:**

Right ventricular strain recorded with CMR serves as a novel indicator to quantify myocardial deformation. Although several studies have reported the predictive value of right ventricular strain determined using CMR, their validity is limited by small sample size and low event number.

**Methods:**

Embase, Medline and Web of Science were searched for studies assessing the prognostic value of myocardial strain. The primary endpoint was a composite of all-cause mortality, cardiovascular death, aborted sudden cardiac death, heart transplantation and heart failure admissions.

**Results:**

A total of 14 studies met the selection criteria and were included in the analysis (n = 3239 adults). The random-effects model showed the association of parameters of right ventricular strain with major adverse cardiac events. Absolute value of right ventricular global longitudinal strain was negatively correlated with right ventricular ejection fraction (hazard ratio: 1.07, 95% confidence interval: 1.05–1.08; *p* = 0.013). Despite the small number of studies, right ventricular radial strain, right ventricular circumferential strain and right ventricular long-axis strain displayed potential prognostic value.

**Conclusions:**

Right ventricular strain measured with CMR is an effective prognostic indicator for cardiovascular disease.

## 1. Introduction

Right ventricular function has emerged as a crucial parameter in the early diagnosis and prognostic assessment of cardiovascular disease [1]. For example, this function has a potential predictive role in pulmonary hypertension (PH) with right ventricular involvement, advanced heart failure (HF) with biventricular involvement [2] and global heart involvement, such as myocardial amyloidosis, as well as in patients surgically treated for tetralogy of Fallot (TOF).

Cardiac magnetic resonance (CMR) is currently the gold standard for assessing right ventricular function [3]. Moreover, right ventricular strain analysis using CMR is one of the several methods for assessing right ventricle (RV) systolic function and detects both myocardial deformability and early abnormalities to provide independent prognostic information. Studies have demonstrated the significance of left ventricular strain in predicting the prognosis of cardiovascular disease [4, 5]. Recently, certain studies have established that RV strain is an independent prognostic factor for several cardiovascular diseases [1, 2]; however, its clinical significance is limited because of the small sample size and the small number of endpoint events. Hence, a systematic review and meta-analysis was conducted to evaluate the prognostic value of right ventricular strain in cardiovascular disease.

## 2. Materials and Methods

### 2.1 Search Strategy

This systematic review and meta-analysis was designed and conducted according to the PRISMA statement (Preferred Reporting Items for Systematic Reviews and Meta-Analyses) [6] and the Cochrane Handbook for Systematic Reviews of Interventions [7]. Two reviewers (BKR and ZZ) systematically searched Embase, Medline and Web of Science databases for eligible studies on CMR strain in patients with cardiovascular diseases. This search was performed using three sets of keywords in combination. The first set included the terms ‘prognostic’ OR ‘prognosis’ OR ‘predictor’ OR ‘outcome’ OR ‘outcomes’. The second set included the terms ‘tissue tracking’ OR “feature tracking’ OR ‘strain’ OR ‘CMR-FT’. The third set included the terms ‘cardiac magnetic resonance’ OR ‘CMR’. The complete search strategy is presented in **Supplementary Table 1**. The results of randomized controlled trials, cohort studies and studies published in peer-reviewed journals were included, and the reference lists of these articles were carefully examined. This study was prospectively registered with the PROSPERO database of systematic reviews (Cardiac magnetic resonance right ventricular strain in predicting prognosis of patients with cardiovascular disease: A systematic review and meta-analysis CRD42021245484).

### 2.2 Inclusion and Exclusion Criteria and Endpoint

Those studies in which patients with cardiovascular disease underwent CMR to assess myocardial strain were included. At least one of the following outcome measures was used to assess prognosis: all-cause mortality, cardiovascular death, aborted sudden cardiac death, heart transplantation and HF admissions. Studies in which patients had undergone surgery or intervention, those that only investigated left heart or right atrial strain and those involving only ultrasound or nuclear medicine were excluded. Conference abstracts, case reports, editorials and commentaries were also excluded.

### 2.3 Study Selection and Quality Evaluation

Studies included in the meta-analysis were independently assessed by two reviewers. They independently reviewed all titles and abstracts and selected eligible articles based on the inclusion and exclusion criteria. Any disagreement was resolved via discussion and submission of the study report to a third reviewer (GYF). Full texts of eligible articles were reviewed.

The Newcastle–Ottawa scale (NOS) [8] was used to systematically evaluate the quality of studies. This scale assesses study quality based on three aspects: selection and definition of included populations (0–4 points), comparability of controlled studies (0–2 points) and determination of results (0–3 points).

### 2.4 Data Extraction

The same two researchers performed independent extraction and review; all disagreements were mutually discussed and resolved by consensus. The following information was extracted from each study: title; authors; publication year; study design type; number of patients; type of disease; clinical information; software used for strain analysis; key CMR results pertaining to the strain, namely, left ventricular ejection fraction (LVEF) and right ventricular ejection fraction (RVEF); and effect size estimation. Categorical data were expressed as percentages, and continuous variables were expressed as mean with standard deviation or median with interquartile range. Effect size estimates were used to extract hazard ratios (HRs) and their 95% confidence intervals (CIs), if available. For grouped data, mean and standard deviation of the groups were combined according to the formula specified by the Cochrane Collaboration.

By convention, negative strain values represent shortening; thus, a higher absolute value (more negative) for global longitudinal strain (GLS) is referred to as ‘better’ and a lower absolute value (less negative or closer to zero) as ‘worse’ [9]. To avoid variability in reporting and interpreting studies in this review, the percentage of GLS was used to indicate a negative sign. Similarly, the percentage of global circumferential strain (GCS) referred to a negative sign, and the percentage of global radial strain (GRS) referred to a positive sign.

### 2.5 Data Analysis

Pooled HRs and their 95% CIs were calculated for the parameters of RV longitudinal strain (RVGLS), RV circumferential strain (RVGCS), RV radial strain (RVGRS), LVEF and RVEF using the random-effects model to ensure consistency. Heterogeneity was assessed using the Cochrane test and discordance factor ($I^{2}$). Sensitivity analyses were performed to assess the robustness of results by re-running the analysis, excluding one study at a time. Simultaneously, a meta-regression was performed for each risk factor to determine the possible factors associated with heterogeneity. STATA (version 16, ICI Stata Corporation, College Station, TX, USA) was used for statistical analysis with two-tailed *p*-values. A *p*-value of <0.05 was considered significant. Correlation analysis among RVGLS, RVEF and RVEDVi (end-diastolic volume index) was performed using STATA.

### 2.6 Patient and Public Involvement in the Study

It was not possible to involve either patients or the public in the design, conduct, reporting or dissemination plans of our research.

## 3. Results

### 3.1 Selection of Eligible Studies 

**Fig. 1.**
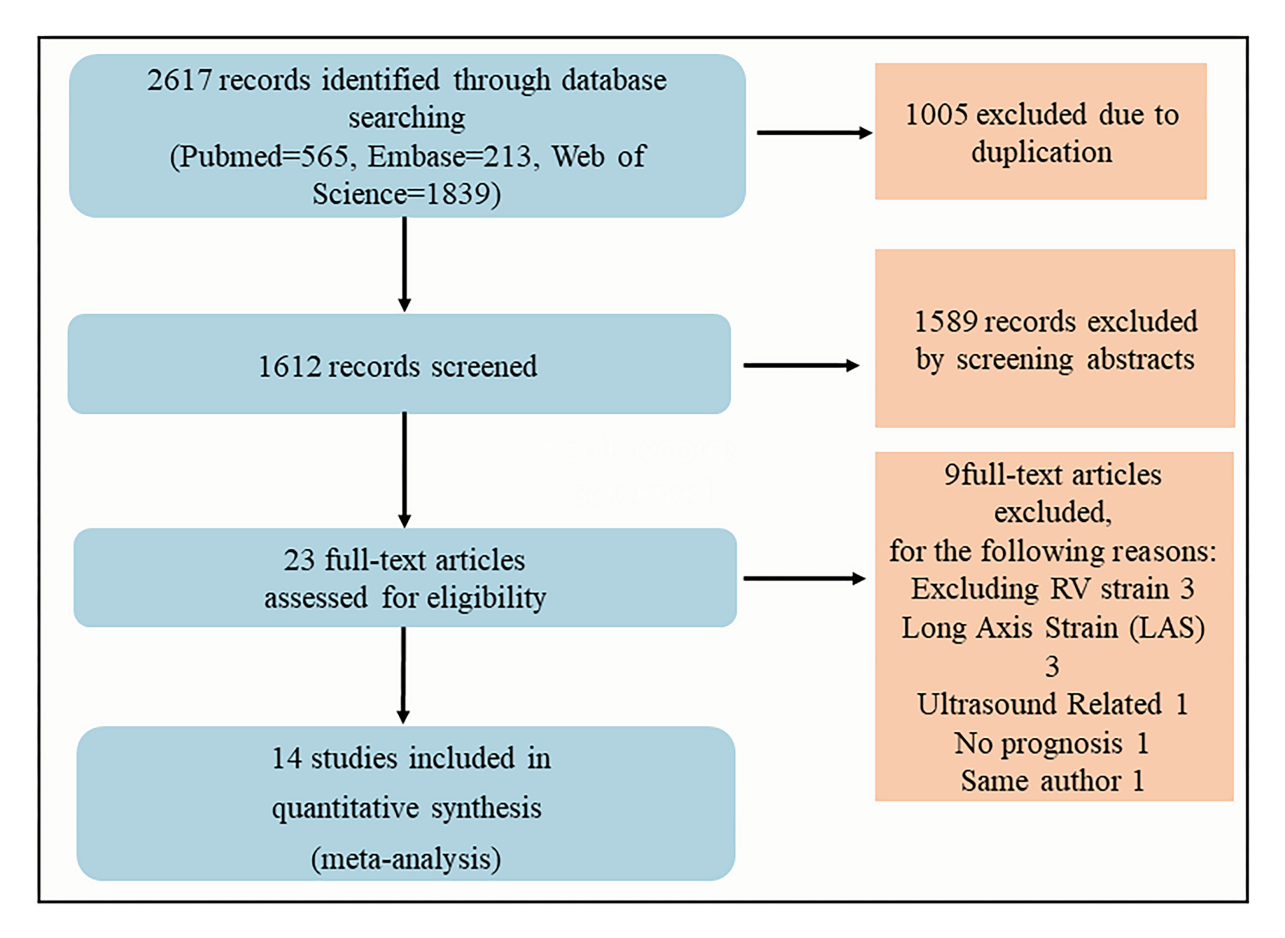
**Search strategy to identify eligible studies by searching databases**.

A total of 2617 relevant abstracts of full-text articles were retrieved. These abstracts included 1005 duplicate articles; 680 articles that only involved left heart and right atrial strain and 180 articles in which prognosis was not covered were excluded. In addition, 205 reviews, abstracts, cases and editorials were excluded. Another 524 records were excluded as these involved animal experiments or comprised only ultrasound, nuclear medicine and surgical and interventional medical history. The remaining 23 articles were full-text reviews, of which 9 were excluded owing to the lack of our pre-specified results, thus leaving 14 articles for detailed analysis [2, 10, 11, 12, 13, 14, 15, 16, 17, 18, 19, 20, 21, 22]. The corresponding author of two studies [11, 13] was same; however, both were included because of differences in parameters, time of data collection and number of patients. The detailed flowchart of the search strategy is shown in Fig. 1.

### 3.2 Patient Characteristics

The study and patient characteristics are listed in Table 1 (Ref. [2, 10, 11, 12, 13, 14, 15, 16, 17, 18, 19, 20, 21, 22]). The information obtained from CMR is presented in Table 2 (Ref. [2, 10, 11, 12, 13, 14, 15, 16, 17, 18, 19, 20, 21, 22]). Our study included ten prospective studies [2, 10, 11, 12, 14, 15, 18, 19, 20, 21] and four retrospective studies [13, 16, 17, 22]. Strain values were calculated using CMR-FT post-processing software; however, the software used in each study varied. Of these, QStrain software (five studies [10, 11, 15, 18, 19]) and CVI42 software (six studies [2, 12, 13, 20, 21, 22]) were commonly used. The total number of subjects included in these 14 studies [2, 10, 11, 12, 13, 14, 15, 16, 17, 18, 19, 20, 21, 22] was 3239; their mean age was 58.9 years, and 67.5% were men. Of these, 37.5% had ischemic cardiomyopathy, 23.5% had dilated cardiomyopathy (DCM) or HF, 9.5% had hypertrophic cardiomyopathy, 8.6% had amyloidosis, 6.4% had PH, 4.0% had arrhythmogenic right ventricular cardiomyopathy (ARVC) and 10.5% had other heart diseases.

**Table 1.** **Characteristics of eligible studies in the meta-analysis**.

	First author	Year	Design	Median follow-up, years	Population Available	Population	Endpoint Included in Analysis	Age, years	Male, %	BMI	Comorbidities, %Coronary artery disease	Comorbidities, %Hypertension	Comorbidities, %Diabetes	Comorbidities, %Dyslipidemia	NOS
predictive value for right ventricular strain:	T Liu [2]	2020	prospective	1.6	192	DCM	MACEs	53 ± 14	72.9	26 ± 5	NA	41.3	14.8	NA	8
	Kazimierczyk [10]	2020	prospective	1.4	28	PH	MACEs	50 ± 16	83.0	NA	NA	NA	NA	NA	5
	Wan [11]	2020	prospective	3.1	129	AL Amyloidosis	all-cause mortality	58 ± 11	61.2	22 ± 3	NA	NA	NA	NA	6
	Li [12]	2020	prospective	1.2	87	AL amyloidosis	all-cause mortality	57 ± 9	64.4	NA	NA	NA	NA	NA	7
	H Liu [13]	2019	retrospective	1.6	64	AL Amyloidosis	all-cause mortality	58 ± 13	56.3	NA	0.0	1.6	3.1	NA	5
	Houard [14]	2019	prospective	4.7	266	HF	Overall death, CV death	60 ± 14	71.0	26 ± 5	51.0	46.0	24.0	NA	8
	Vos [18]	2022	prospective	8.0	33	PH	MACEs	58 (46–72)	24.0	NA	NA	NA	NA	NA	7
	Cittar [20]	2021	prospective	1.6	273	NIDCM	MACEs	51 (41–60)	66.0	NA	NA	35.0	16.0	NA	6
	Stiermaier [21]	2020	prospective	1.0	1235	STEMI/NSTEMI	MACEs	64 (53–73)	74.9	NA	100.0	71.9	23.4	38.3	8
	Yang [16]	2016	retrospective	1.2	364	Consecutive series	MACEs	66	65.0	23 ± 3	28.0	58.0	22.0	0.4	6
	Siqueira [17]	2016	retrospective	2.0	103	PH	MACEs	52 ± 12	26.4	NA	NA	21.8	10.9	15.5	7
No predictive value for right ventricular strain:	Padervinskienė [15]	2019	prospective	2.5	43	PH	MACEs	55	35.0	NA	NA	NA	NA	NA	7
	Bourfiss [19]	2022	prospective	4.3	132	ARVC	the occurrence of sustained VA following CMR	41 ± 16	43.0	NA	NA	NA	NA	NA	8
	Mahmod [22]	2022	retrospective	4.4	290	HCM	MACEs	52 ± 15	74%	28 ± 5	NA	30	78	NA	7

PH, Pulmonary hypertension; HF, heart failure; AL, amyloid light-chain; STEMI, ST-elevation myocardial infarction; NSTEMI, Non-ST-elevation myocardial infarction; DCM, Dilated cardiomyopathy; NIDCM, Non-ischemic dilated cardiomyopathy; ARVC, Arrhythmogenic right ventricular cardiomyopathy; HCM, Hypertrophic cardiomyopathy; CV, Cardiovascular; MACE, Major Adverse Cardiovascular Events; BMI, Body Mass Index; NOS, Newcastle-Ottawa Scale for quality assessment of non-randomized studies; values are mean ± SD (%).

**Table 2.** **Information obtained from CMR in the studies**.

	First Author	Vendor	Software	LVEF (%)	Univariable analysis: HR, CI, *p value*	RVEF (%)	Univar able analysis: HR, CI, *p value*	RVEDV	Univariable analysis: HR, CI, *p value*	RVEDVi	Univariable analysis: HR, CI, *p value*	Strain	RVGLS	Univariable analysis: HR, CI, *p value*	Multivariable analysis: HR, CI, *p value*	RVGRS	Univariable analysis: HR, CI, *p value*	RVGCS	Univariable analysis: HR, CI, *p value*
predictive value for right ventricular strain	T Liu [2]	Siemens GE	CVI42 software	22.4 ± 9.8	0.96 (0.91–1.02), *p* = 0.169	30.4 ± 14.1	0.99 (0.96–1.02) *p* = 0.686	133 ± 44	1.01 (1–1.02) *p* = 0.01	NA	NA	L/C	–10.5 ± 5.2	1.14 (1.01–1.28) *p* = 0.035	1.17 (1.04–1.32) *p* = 0.01	NA	NA	–7.7 ± 3.8	1.05 (0.93–1.19) *p* = 0.456
	Kazimierczyk [10]	Siemens	Qstrain software	60.3 ± 9.9	1.05 (0.96–1.15), *p* = 0.23	25.8 ± 13.6	0.88 (0.78–0.99) *p* = 0.03	NA	NA	118.2 ± 21.7	1.01 (0.98–1.03) *p* = 0.48	L	–16.2 ± 8.1	1.11 (1.01–1.31) *p* = 0.04	NA	NA	NA	NA	NA
	Wan [11]	Siemens	Qstrain software	47.0 ± 15.0	0.98 (0.96–0.99), *p *< 0.001	47.0 ± 14.0	0.96 (0.95–0.98) *p *< 0.001	NA	NA	68 ± 23	1.004 (0.995–1.013) *p* = 0.362	L	–14.2 ± 7.0	1.10 (1.06–1.14) *p *< 0.001	1.07 (1.03–1.11) *p* = 0.001	NA	NA	NA	NA
	Li [12]	Siemens	CVI42 software	58.4 ± 10.7	0.94 (0.91–0.97), *p *< 0.001	57.2 ± 10.2	0.96 (0.93–0.99) *p* = 0.005	NA	NA	63.3 ± 14.5	NA	L/R/C	–19.1 ± 6.3	1.18 (1.10–1.26) *p *< 0.001	1.10 (1.00–1.21) *p* = 0.047	22.4 ± 7.1	0.95 (0.90–1.00) *p* = 0.048	–13.3 ± 4.4	NA
	H Liu [13]	Siemens	CVI42 software	52.2 ± 12.6	0.97 (0.95–0.99), *p* = 0.001	41.5 ± 9.9	0.99 (0.97–1.01) *p* = 0.097	NA	NA	61.1 ± 20.5	NA	L/R/C	NA	1.12 (1.03–1.22) *p* = 0.006	1.02 (0.89–1.16) *p* = 0.776	NA	0.92 (0.88–0.96) *p *< 0.001	NA	1.02 (0.89–1.16) *p* = 0.776
	Houard [14]	Philips	Segment version 2.2	23 ± 7	0.98 (0.90–1.01), *p* = 0.16	42.0 ± 15.0	0.98 (0.97–0.99) *p* = 0.03	NA	NA	86 ± 33	NA	L	–11.8 ± 4.3	1.06 (1.01–1.11) *p* = 0.015	1.05 (0.99–1.10) *p* = 0.05	NA	NA	NA	NA
	Vos [18]	Siemens Philips, GE	Qstrain software	58.0 ± 9.0	NA	46.0 (38.0–53.0)	0.96 (0.92–1.01) *p* = 0.14	NA	NA	101 (86–138)	1.01 (1–1.02) *p* = 0.01	L/C	–20.0 ± 6.0	1.18 (1.04–1.34) *p* = 0.01	NA	NA	NA	–12.0 ± 5.0	1.01 (0.91–1.13) *p* = 0.80
	Cittar [20]	Siemens Philips	CVI42 software	34.0 (25.0–43.0)	1.08 (1.04–1.11), *p *< 0.001	51.0 (40.0–59.0)	1.05 (1.03–1.08), *p *< 0.001	NA	NA	NA	1.02 (1.1–1.03) *p *< 0.001	L/C/R	–19.1 (–15.4 to –23.0)	1.06 (1.02–1.10) *p* = 0.001	NA	17.6 (12.0–23.7)	NA	10.5 (–7.5 to –13.2)	NA
	Stiermaier [21]	NA	CVI42 software	50.6 (43.5–57.5)	NA	61.3 (54.2–67.8)	NA	NA	NA	NA	NA	L	–21.3 (–16.3 to 26.1)	1.07 (1.04–1.10) *p *< 0.001	NA	NA	NA	NA	NA
	Yang [16]	GE, Philips	2D Cardiac Performance Analysis	48.0 ± 20.0	0.96 (0.94–0.98), *p *< 0.0001	44.9 ± 11.3	0.94 (0.92–0.97) *p *< 0.0001	NA	NA	66.4 (56.1–80.5)	1 (0.99–1.01) *p* = 0.085	L/R/C	–18.3 ± 6.9	1.03 (0.98–1.09) *p* = 0.1708	NA	21.0 ± 8.0	0.92 (0.87–0.96) *p* = 0.0010	–12.2 ± 3.8	1.11 (1.01–1.23) *p* = 0.0253
	Siqueira [17]	Simens Philips	2D CPA MR	58.9 ± 9.8	NA, *p *< 0.001	42.2 ± 13.7	*p *< 0.001	NA	NA	98.8 (75.3–127)	NA	L/C/	–15.9 ± 6.4	NA	NA	NA	*p* = 0.001	–11.2 ± 4.9	*p* = 0.007
no predictive value for right ventricular strain:	Padervinskienė [15]	Siemens	Qstrain software	56.0 ± 13.2	NA	38.2 ± 12.9	NA	NA	NA	NA	NA	L	NA	NA	NA	NA	NA	NA	NA
	Bourfiss [19]	Siemens Philips,GE	Qstrain software	56.0 ± 8.0	NA		NA	NA	NA	102 ± 30	NA	L	–22.5 ± 8.4	1.05 (1.00–1.11) *p* = 0.053	NA	NA	NA	NA	NA
	Mahmod [22]	Avanto and TIMTrio, Siemens GE	cvi42	70.0 ± 6.0	NA	63.0 ± 7.0	1.07 (1.05–1.10) *p *< 0.001	52 ± 42	NA	NA	NA	L	–21.0 ± 5.0	1.03 (1.00–1.06) *p* = 0.096	NA	17.0 ± 7.0	NA	–10.0 ± 4.0	NA

Values are mean ± SD (%); HR, Hazard ratio; CI, Confidence interval; LVEF, Left ventricular ejection fraction; RVEF, Right Ventricular Ejection Fraction; EDV, end-diastolic volume indexed; EDVi, end-diastolic volume indexed; RVGLS, right ventricle longitudinal strain; RVGCS, right ventricle circumferential strain; RVGRS, right ventricle radial strain.

### 3.3 Outcomes

**Fig. 2.**
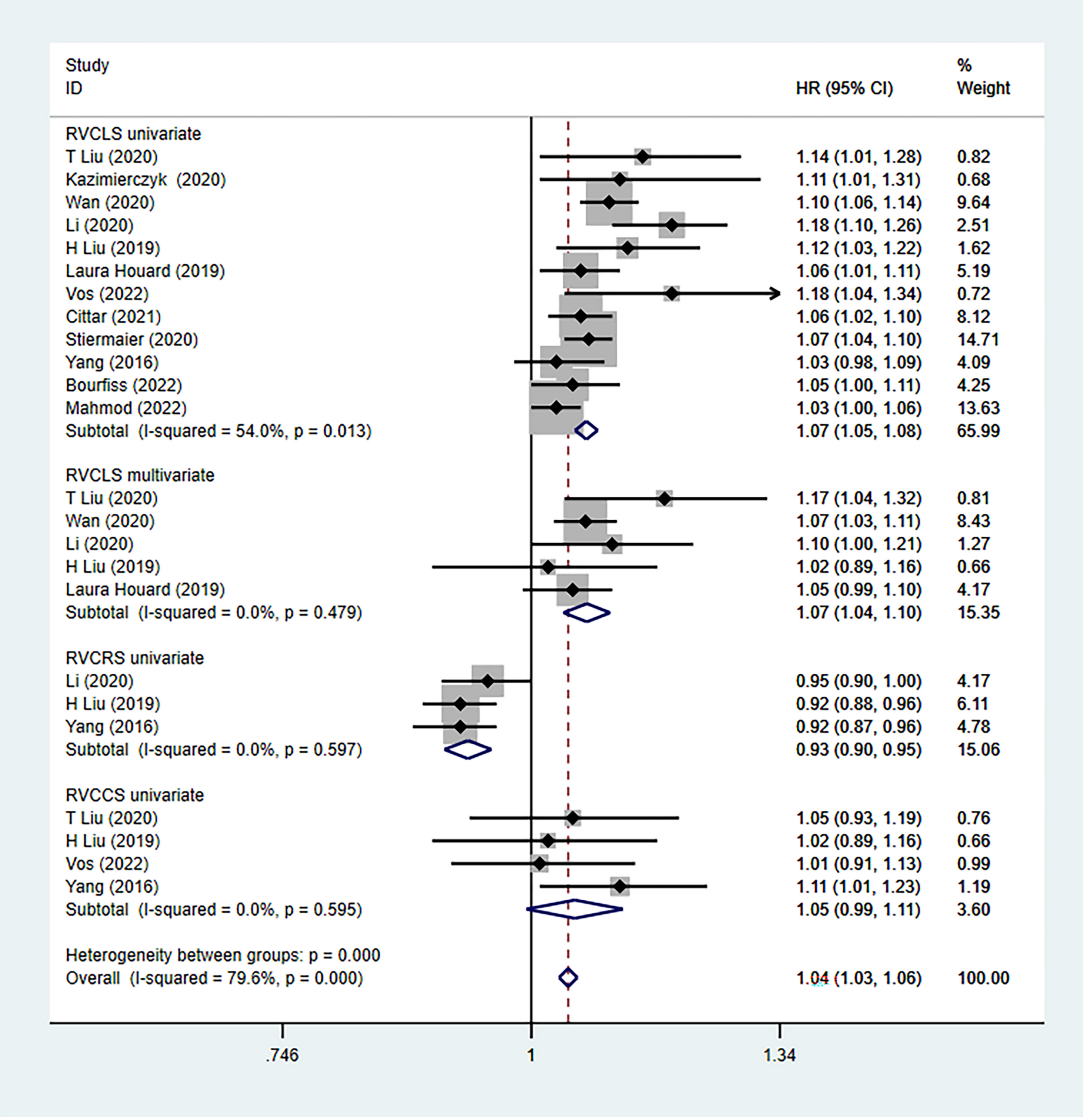
**Relationship among RVGLS, RVGRS and RVGCS, as determined with univariable and multivariable analyses**. RVGLS, RV longitudinal strain; RVGRS, RV radial strain; RVGCS, RV circumferential strain.

**Fig. 3.**
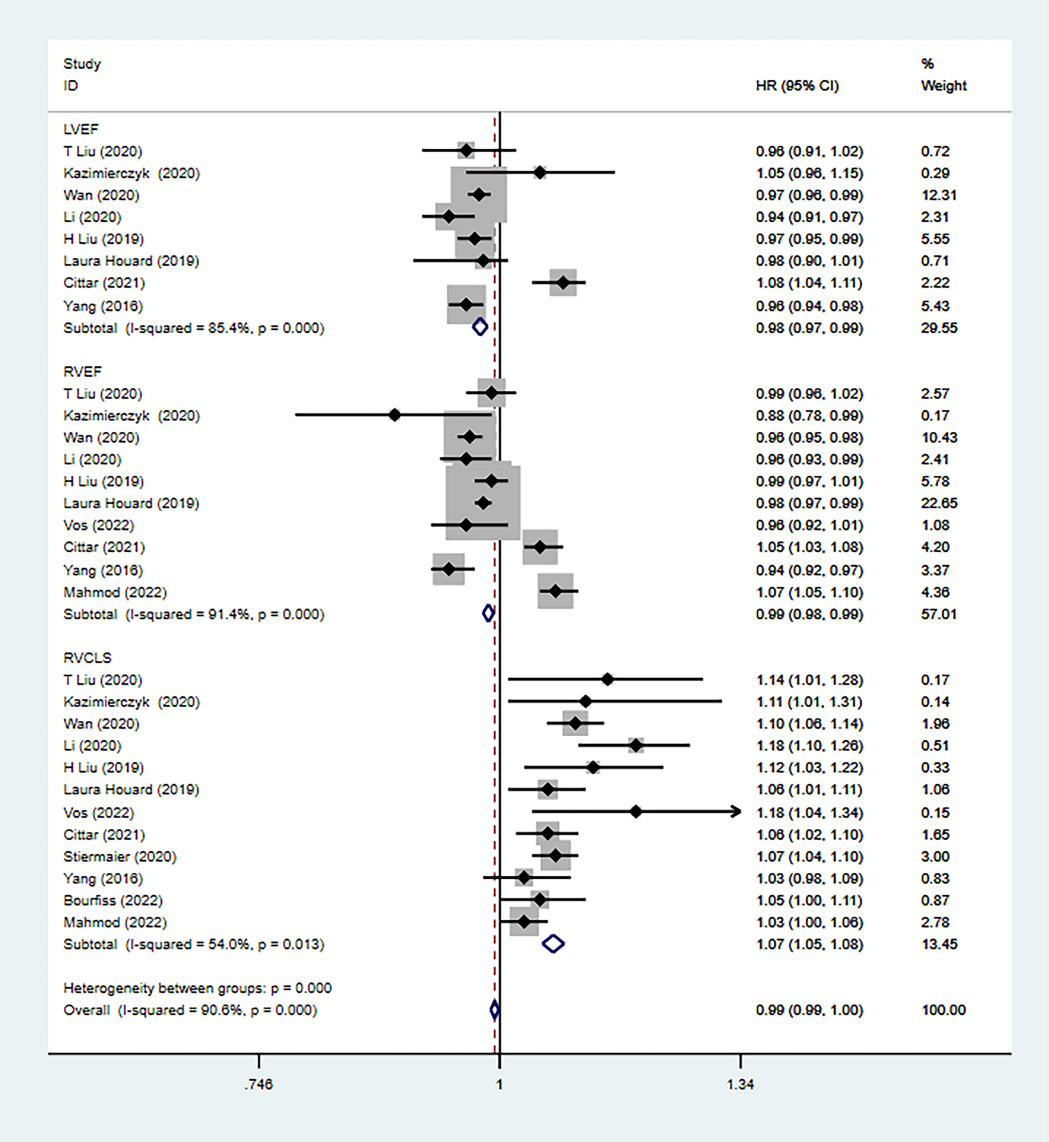
**Comparison of the predictive values of RVGLS, LVEF and RVEF**. RVGLS, RV longitudinal strain; LVEF, Left ventricular ejection fraction; RVEF, right ventricular ejection fraction.

**Fig. 4.**
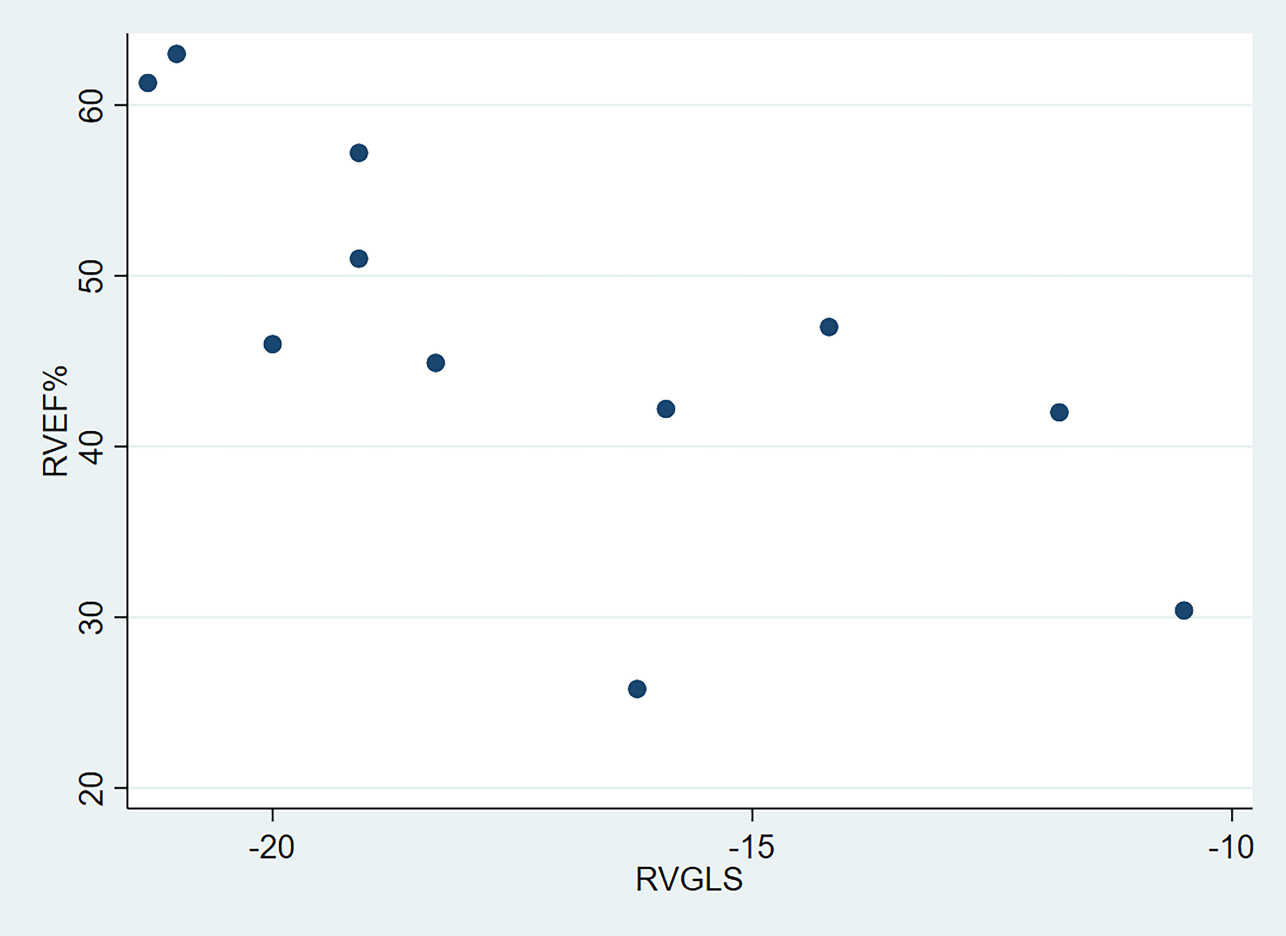
**Significant negative correlation of RVEF with RVGLS**. RVEF, right ventricular ejection fraction; RVGLS, RV longitudinal strain.

The cut-off values of RVGLS obtained in five studies [2, 13, 17, 19, 20] were –8.5%, –17%, –15%, –19.1% and –22.5%. Univariable analysis was performed in twelve studies [2, 10, 11, 12, 13, 14, 16, 18, 19, 20, 21, 22] and multivariable analysis in five studies [2, 11, 12, 13, 14] on the prognostic value of RVGLS after adjusting for significant factors. In the univariate analysis, the pooled risk (HR) of RVGLS calculated using the random-effects model was 1.07 (95% CI: 1.05–1.08; *p* = 0.013). In other words, for each 1% decrease in RVGLS, the risk of major adverse cardiovascular event (MACE) occurrence increased by 7% (Fig. 2). Moderate heterogeneity was detected ($I^{2}$ = 54%). In the multivariate analysis, the pooled risk (HR) of RVGLS estimated using the random-effects model was 1.07 (95% CI: 1.04–1.10; *p* = 0.48) (Fig. 2). There were three univariate analysis studies for RVGRS [12, 13, 16] and four [2, 13, 16, 18] for RVGCS. The relationship among RVGRS, RVGCS and MACE was analysed separately. The pooled risk (HR) of RVGRS was 0.93 (95% CI: 0.90–0.95; *p* = 0.479) and that of RVGCS was 1.05 (95% CI: 0.99–1.11; *p* = 0.595) in the random-effects model, with no heterogeneity (Fig. 2). Furthermore, the prognostic value of ejection fraction was determined in the included studies. The pooled risk (HR) was 0.98 (95% CI: 0.97–0.99; *p *< 0.001; $I^{2}$ = 85.4%) for LVEF and 0.99 (95% CI: 0.98–0.99; *p* = 0.075; $I^{2}$ = 91.4%) for RVEF (Fig. 3). Both were calculated using the random-effects model. However, in univariate analysis, the heterogeneity of LVEF and RVEF was clearly increased compared with RVGLS. Pearson’s correlation analysis showed a significantly negative correlation between RVEF and RVGLS (r = –0.721, *p* = 0.012; Fig. 4). However, there was no significant correlation between RVGLS and RVEDVi (r = –0.708, *p =* 0.075).

In addition, two articles on the prognostic value of right ventricular long axis strain (RV-LAS) were retrieved to investigate its ability to assess prognosis in patients with hypertrophic cardiomyopathy (HCM) and non-ischemic dilated cardiomyopathy (NIDCM) [23, 24]. Because the number of articles was too small, the corresponding meta-analysis was not performed.

### 3.4 Publication Bias and Heterogeneity

Because of the small number of articles included in the meta-analysis, corresponding publication bias analysis was not performed. In the meta-regression analysis, 14 of the hypothesized confounding factors, such as publication year, follow-up time, age, sample size and gender, were observed to be associated with the variability of RVGLS (**Supplementary Table 2**). In addition, no confounding factors could significantly explain the heterogeneity of RVGLS. In sensitivity analysis, the articles were excluded one by one, which revealed that Yang *et al*.’s [16] and Mahmod *et al*.’s [22] studies exerted an immense negative impact on the combined HR results (**Supplementary Fig. 1**). A possible reason could be that Yang included consecutive patients in their CMR examinations. Moreover, specific diseases were not studied and distinguished, which resulted in a large heterogeneity. Mahmod *et al*. [22] defined composite cardiovascular events as non-sustained ventricular tachycardia (NSVT), stroke, HF hospitalisation and cardiovascular death. This definition might have affected the final conclusion because there were multiple outcome events. Furthermore, Mahmod *et al*. [22] demonstrated that strain in the right ventricle is of great value in predicting the development of NSVT. However, these studies [16, 22] were included in the meta-analysis because of the predictive value of right ventricular strain in its findings.

## 4. Discussion

This meta-analysis confirmed that RV strain is an important independent predictor of adverse outcomes in many cardiovascular diseases and that RVGLS assessed with CMR has significant prognostic significance in patients with various underlying cardiac abnormalities. In addition, RVGLS values decreased with decreasing RVEF values, which indicates a significant correlation between the two. Also, RVGRS and RVGCS appear to have good predictive value although the number of studies in this analysis is too small to confirm this finding. Finally, RV-LAS was observed to be a powerful predictor of cardiovascular disease development.

CMR has high temporal and spatial resolution, can perform gracilis imaging well and is the gold standard for right ventricular structural evaluation in clinical studies [3]. Two-dimensional echocardiography is the most widely used imaging modality in RV assessment; however, its main limitation is that the image quality depends on operator experience and subject characteristics. EF is the most commonly used and key index that shows systolic function in clinical practice. Nevertheless, EF reflects only global volume changes and cannot reflect alterations in myocardial regional motion or impaired early diastolic function. Myocardial strain technique is a non-invasive quantitative analysis of global and regional myocardial systolic and diastolic functions. Speckle tracking echocardiography (STE) is an accurate and simple method to assess myocardial strain. Park *et al*. [25] showed that RVGLS obtained via two-dimensional STE correlates well with RVEF and longitudinal strain obtained with CMR. Although STE is primarily a post-processing method, it requires a specific frame rate (50–70 frames/s) and high image quality during image acquisition [26]. Moreover, its wide applicability may be hindered by poor acoustic windows. CMR plays an important role in assessing myocardial strain processes; for example, GCS exhibits better repeatability when calculated using CMR [27].

**Fig. 5.**
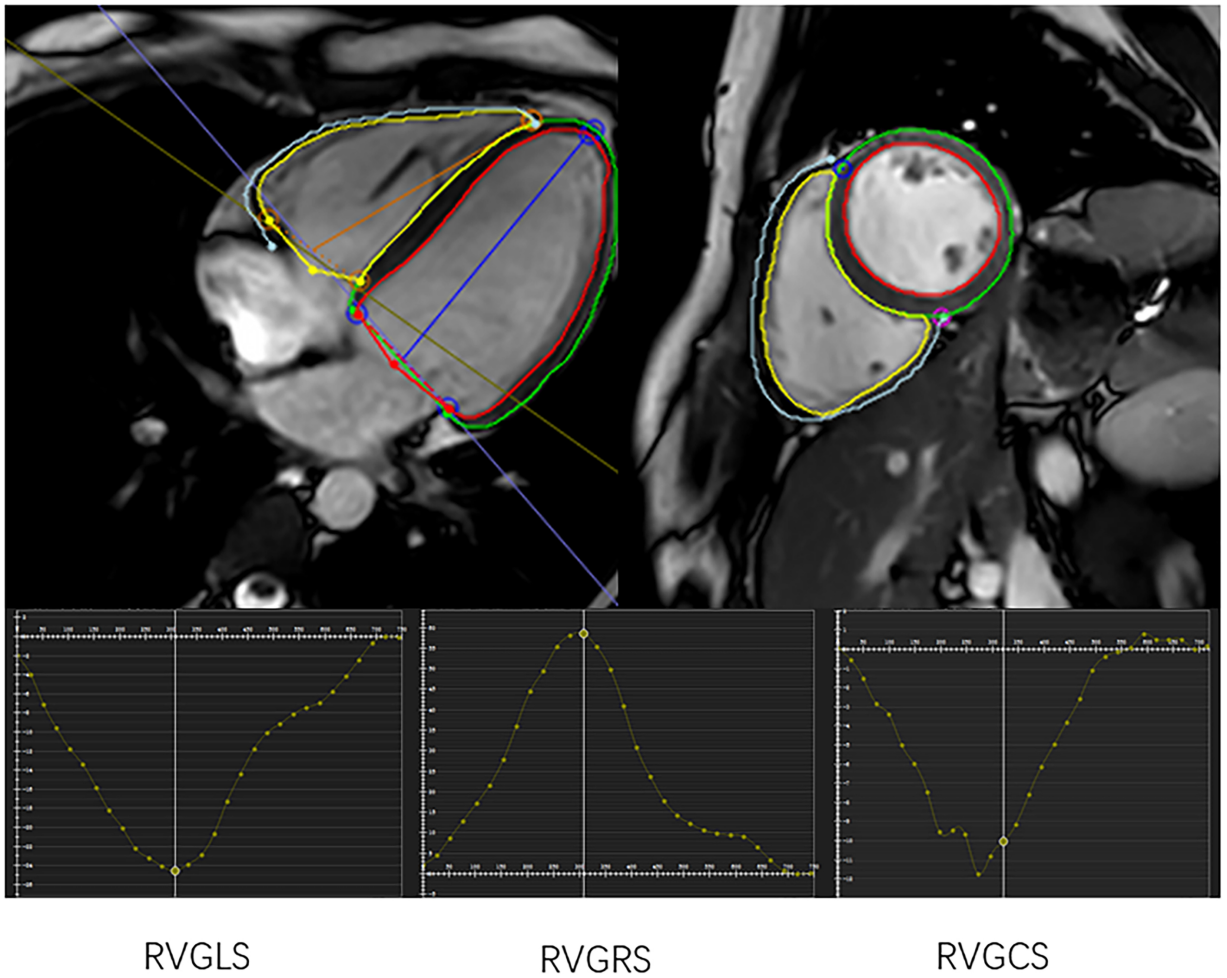
**Three-dimensional FT global longitudinal strain (GLS), global radial strain (GRS) and global circumferential strain (GCS) of RV obtained using CVI42**.

Several methods are available for obtaining information on myocardial deformation using CMR, which are broadly divided into two categories. The first category requires additional scans and includes CMR tagging, displacement encoding with stimulated echoes (DENSE) and strain-encoded imaging (SENC). The second category is CMR Feature tracking (CMR-FT), which records myocardial strain by acquiring retrospective CMR cine images [27]. CMR tagging, a gold standard for measuring myocardial strain, works on the principle of superimposing magnetic tags (black lines and tags) orthogonally onto the myocardium at the beginning of the cine sequence. The deformation of these lines throughout the cardiac cycle is subsequently analysed [28]. Although it is the most effective CMR technique for assessing myocardial strain [29], its use is limited owing to label fading and low spatial resolution, which reduce its accuracy [28]. CMR tagging requires certain additional sequences, prolonged image acquisition and breath-hold time. Furthermore, it is insensitive to through-plane motion [30]. DENSE for displacement coding was first introduced in 1999 [31], and it is a technique for encoding tissue displacement into the phase of an image. DENSE possesses high temporal and spatial resolution as well as superior strain accuracy and reproducibility [32]. However, additional sequences cause prolonged scanning times, thereby limiting its clinical application. SENC was developed based on the concept of CMR tagging [33], thus enabling the quantification of local deformation of tissues. To calculate myocardial strain, SENC uses magnetized tags parallel to the image plane (rather than being orthogonal as in CMR tagging), which allows higher spatial resolution and, hence, better right ventricular endocardial delineation. Therefore, this technique is effective in quantifying the strain in the through-plane. Because T1 relaxation time leads to fading of the tag, SENC cannot be used to assess myocardial deformation throughout the cardiac cycle. CMR-FT is a simple and convenient new technique for determining myocardial deformation, which can quantitatively analyse the systolic and diastolic functions of the global and regional myocardium in a non-invasive manner. This technology uses post-processing software to delineate the relative motion and displacement of voxels on the endocardial and epicardial boundaries of the right ventricle during the cardiac cycle. Moreover, local or global strain and strain rate of the right ventricle are obtained in radial, circumferential and longitudinal directions. Currently, CMR-FT post-processing analysis can be accomplished with several commonly used commercial software, such as TomTec, Circle, Medis and Medviso. In the case of CVI42, RV myocardial strain is estimated by loading images from four-chamber and short-axis slices into the strain analysis module. In all series, endocardial and epicardial contours are manually delineated per slice at end-diastole. Global myocardial strain parameters are obtained automatically [34], while Fig. 5 shows the specific measurement method. However, because the strain is not derived from the full thickness of the myocardial tissue, CMR-FT may be associated with low accuracy and measurement variability. As additional breath-hold scanning sequences are not required and the reduced scanning time improves the imaging efficiency, CMR-FT is an attractive clinical method. Therefore, all our included studies utilized this method for strain analysis. Additionally, RV-LAS refers to the percentage change in length between the LV apical adventitial border and the midpoint of the line between tricuspid annulus at the end of systole and that at diastole. Long-axis strain (LAS) is a rapidly derived new parameter in CMR cine images, which can be evaluated online without additional software tools and has good clinical application value.

Unlike left ventricular measurements, the assessment of right ventricular function is challenging because right ventricular trabecular muscles are significantly more in number, right ventricular wall is significantly thinner and arrangement and movement of cardiomyocytes are different [35]. CMR can reveal the morphology and function of the heart and is the gold standard for assessing the complex geometry of RV. Right ventricular strain obtained using CMR is more sensitive than EF and has a high clinical value in assessing right ventricular myocardial damage in patients with subclinical myocardial damage. The method is also useful in risk stratification and in determining the therapeutic effect. Compared with conventional parameters of RV systolic function, such as RVEF, RV strain can detect subtle changes in RV function without significant wall motion abnormalities or reduced global RV systolic function. Such changes have significantly higher predictive values than conventional parameters. For example, Mahmod *et al*. [22] found that both RV and LV strain were impaired in patients with HCM despite normal LVEFd. A small decrease was observed in RVEF, but it was within the acceptable normal range. Moreover, Henning *et al*. [36] found that the percentage of normal myocardium in LV and RV detected using rapid SENC (normal LV and RV myocardial segmental strain ≤–17%) better identifies asymptomatic patients with subclinical LV dysfunction. This technique may be useful in the early identification of healthy subjects who may be at risk for HF as well as in the monitoring of LV and RV deformation during pharmacological interventions in future studies. RV strain can help in detecting the subclinical stage of PH and in assessing the disease and the associated prognosis [17]. In congenital heart diseases, such as TOF, CMR can assess the efficacy and necessity of surgery. Moon *et al*. [37] used CMR-FT and found that right ventricular strain values were significantly lower in the TOF group than in the normal group and that right ventricular longitudinal strain was closely related to adverse outcomes in patients with TOF. Early diagnosis and analysis of wall motion abnormalities are possible in patients with ARVC [38]. CMR-FT strain analysis helps to objectively quantify global/regional right ventricular dysfunction and dyssynchrony in patients with ARVC and provides effective diagnostic information.

The cut-off values of RVGLS were obtained in five studies [2, 13, 17, 19, 20]. Although the strain decreased compared with the normal group, the five values were significantly different. This variation could be attributed to the fact that the sources of patients differed in the five included studies [2, 13, 17, 19, 20]. The study by Liu *et al*. [2] only included patients with HF in stages C and D of cardiac function; hence, RVGLS was relatively worse.

RV fibres are primarily arranged along the longitudinal axis under the epicardium. In healthy individuals, longitudinal shortening largely leads to RV shortening [39]. Twelve studies [2, 10, 11, 12, 13, 14, 16, 18, 19, 20, 21, 22] on RVGLS were included in our meta-analysis, and despite the presence of moderate heterogeneity, the pooled effect values demonstrated the prognostic value of RVGLS. Of these, three articles concluded that right ventricular strain did not have a statistically significant prognostic value in cardiovascular disease. The following could be the reasons for this conclusion: Yang *et al*. [16] consecutively included patients with CMR findings and did not classify the disease specifically; hence the heterogeneity was relatively high. Bourfiss *et al*. [19] found that RV and LV strain were no longer significant predictors of persistent ventricular arrhythmias (VA) after adjusting for risk factors, such as RVEF and LVEF. The reason could be that most patients who developed VA already had advanced structural disease, but decreased RV longitudinal and LV circumferential strain were observed in other patients with ARVC and persistent VA during follow-up, thus indicating that strain can reflect changes in ventricular function. Although Mahmod *et al*. [22] noted that the decrease in RVGLS was not associated with cardiovascular composite events, it had a significant predictive value for the occurrence of non-sustained ventricular tachycardia. Several patients in the included studies had right heart involvement and global involvement, but a certain bias existed. Hence, the additional independent prognostic value of right ventricular strain must be carefully studied while considering conventional cardiac function. In four other studies [2, 13, 16, 18] that included RVGCS, it was shown to have good prognostic ability. However, more research on RVGRS and RVGCS is required because of the small number of available studies. In three studies [12, 16, 20], RVGRS exhibited good predictive power, possibly because strict motion of the basal RV and the thin atrioventricular free wall hindered adequate endocardial tracking on long-axis views [16]. The contribution of circumferential strain is important in patients with PH. Unfortunately, data on RVGCS were not included in the three studies [12, 16, 20] on PH, and thus, the results could not be validated. Yang *et al*. [23] showed that the predictive ability of RV-LAS was better than that of RVEF and TAPSE for the poor prognosis of patients with HCM. Arenja *et al*. [24] showed that RV-LAS had the highest diagnostic accuracy in the cohort of patients with NIDCM. Moreover, RV-LAS was an independent marker of MACEs in multivariate analysis. The above studies assert the important role of RV-LAS in prognosis prediction. RV-LAS is defined as the change in length from the LV apex to the tricuspid annulus rather than the RV apex; therefore, it may incorporate both LV and RV longitudinal functions. With the increase in studies on RV-LAS, in the future, the predictive value of RV-LAS can be proved using a meta-analysis.

This systematic review and meta-analysis has certain limitations. First, because studies on right ventricular strain are currently in the developmental stage, the number of studies included in this meta-analysis is relatively small (n = 14). Therefore, publication bias could not be explored. Moreover, only five studies [2, 13, 17, 19, 20] included cut-off values and they dealt with different diseases; hence, a pooled effect size and cut-off value could not be calculated for a specific disease. We are hopeful that with the increase in the number of in-depth studies in this field, we would be able to update and include more studies in the future to provide robust evidence. Furthermore, we believe that we would have a better understanding of the prognostic value of RVGRS and RVGCS. Second, as with meta-analyses of several observational studies, moderate heterogeneity exists among the articles because of differences in the study design, inclusion criteria, follow-up time and vendor software. Although we performed a correlative meta-regression analysis for some measures, we were unable to perform a meta-regression analysis due to the diverse variety of software vendors for the articles we included. it is not well understood whether differences in software suppliers are the source of heterogeneity. Our study demonstrated a significant negative correlation between RVGLS and RVEF. These conclusions have been confirmed in several studies [40, 41]. We understand that strain values may be affected by factors such as magnetic fields and post-processing software. With the increase in the studies on right ventricular strain, in the future, we can analyse the above factors in subgroups. Finally, to reduce the heterogeneity of articles, we excluded studies involving patients with a history of cardiac surgery. Strain is a valuable prognostic indicator for patients with congenital heart disease [42]. With continued technological advancements, a meta-analysis of these patients can be performed in the future.

## 5. Conclusions

This systematic review and meta-analysis establishes the prognostic value of right ventricular strain determined using CMR in cardiovascular disease. RVGLS assessed with CMR has significant prognostic implications in patients with different underlying cardiac abnormalities. RVGRS, RVGCS and RV-LAS appear to show good prognostic value, and although the number of studies on these strains is small, they can pave way for future studies and thus validate our conclusions. In the future, the prognostic value of right ventricular strain in different diseases can be investigated to obtain cut-off values that correspond to different diseases, thereby guiding treatment decisions and prognostic stratification.

## Supplementary Material

Supplementary material associated with this article can be found, in the online version, at https://doi.org/10.31083/j.rcm2312406.
