# Supplementary material for: Prognostic Value of Cardiac Magnetic Resonance in Assessing Right Ventricular Strain in Cardiovascular Disease: A Systematic Review and Meta-Analysis
Source: Rev Cardiovasc Med. 2022 Dec 12;23(12):406. doi: 10.31083/j.rcm2312406 (PMC11270452; doi:10.31083/j.rcm2312406)
Supplement: Supplementary file 1 [file 2153-8174-23-12-406-s1.zip › 2153-8174-23-12-406-s1/v0-Supplementary Materials.docx]

Supplementary Table 1. Searching strategy for locating eligible studies.

| Search number | Query | Sort By | Search Details | Results | Time |
| --- | --- | --- | --- | --- | --- |
| 4 | ((#1) AND (#2)) AND (#3) | Most Recent | ("prognostic"[Title/Abstract] OR "prognosis"[Title/Abstract] OR "predictor"[Title/Abstract] OR "outcome"[Title/Abstract] OR "outcomes"[Title/Abstract]) AND ("tissue tracking"[Title/Abstract] OR "feature tracking"[Title/Abstract] OR "strain"[Title/Abstract] OR "cmr ft"[Title/Abstract]) AND ("cardiac magnetic resonance"[Title/Abstract] OR "CMR"[Title/Abstract]) | 564 | 8:36:39 |
| 3 | (‘cardiac magnetic resonance’[Title/Abstract]) OR (CMR[Title/Abstract]) | Most Recent | "cardiac magnetic resonance"[Title/Abstract] OR "CMR"[Title/Abstract] | 18,981 | 8:36:10 |
| 2 | (((‘tissue tracking’[Title/Abstract]) OR (‘feature tracking’[Title/Abstract])) OR (strain[Title/Abstract])) OR (‘CMR-FT’[Title/Abstract]) | Most Recent | "tissue tracking"[Title/Abstract] OR "feature tracking"[Title/Abstract] OR "strain"[Title/Abstract] OR "cmr ft"[Title/Abstract] | 477,674 | 8:35:48 |
| 1 | ((((prognostic[Title/Abstract]) OR (prognosis[Title/Abstract])) OR (predictor[Title/Abstract])) OR (outcome[Title/Abstract])) OR (outcomes[Title/Abstract]) | Most Recent | "prognostic"[Title/Abstract] OR "prognosis"[Title/Abstract] OR "predictor"[Title/Abstract] OR "outcome"[Title/Abstract] OR "outcomes"[Title/Abstract] | 2,723,258 | 8:35:24 |

Supplementary Table 2. Meta regression.

| Variables | *P* value |
| --- | --- |
| year | 0.904 |
| medianfollowupyears | 0.529 |
| populationavailable | 0.457 |
| ageyears | 0.814 |
| male | 0.339 |


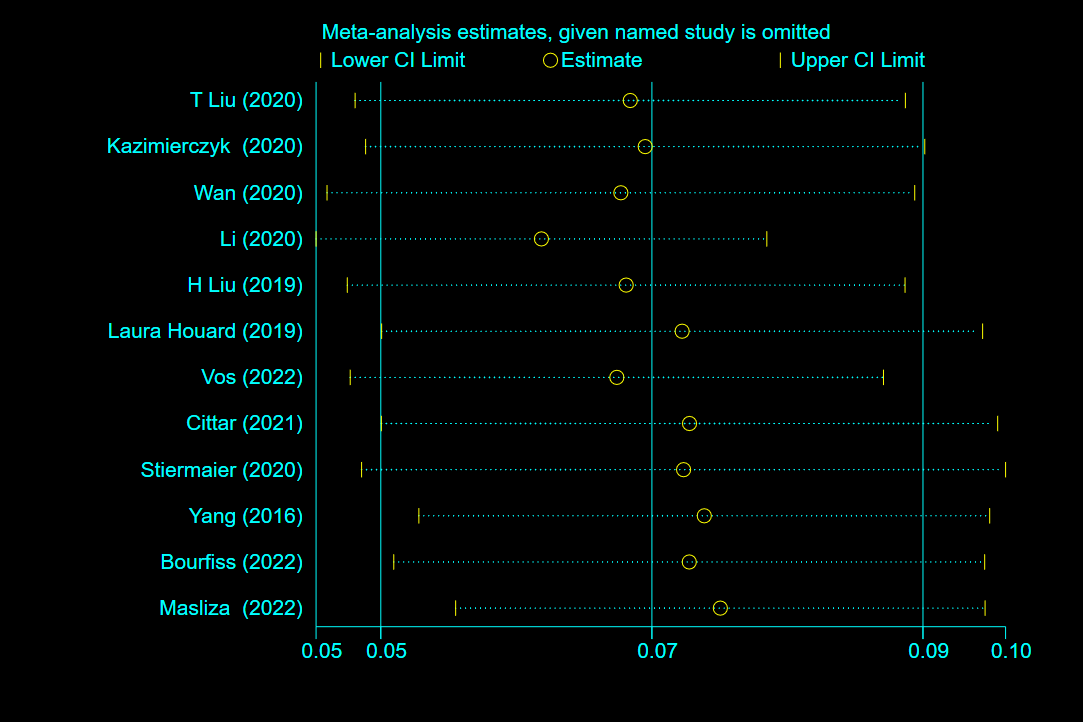


**Supplementary Fig. 1.** **Sensitivity Analyses.**
